# Diets Alter the Gut Microbiome of Crocodile Lizards

**DOI:** 10.3389/fmicb.2017.02073

**Published:** 2017-10-25

**Authors:** Hai-Ying Jiang, Jing-E Ma, Juan Li, Xiu-Juan Zhang, Lin-Miao Li, Nan He, Hai-Yang Liu, Shu-Yi Luo, Zheng-Jun Wu, Ri-Chou Han, Jin-Ping Chen

**Affiliations:** ^1^South China Botanical Garden, Chinese Academy of Sciences, Guangzhou, China; ^2^Guangdong Key Laboratory of Animal Conservation and Resource Utilization, Guangdong Public Laboratory of Wild Animal Conservation and Utilization, Guangdong Institute of Applied Biological Resources, Guangzhou, China; ^3^College of Life Sciences, University of Chinese Academy of Sciences, Huairou, China; ^4^Guangdong Luokeng Shinisaurus crocodilurus National Nature Reserve, Shaoguan, China; ^5^Guangxi Daguishan Crocodile Lizard National Nature Reserve, Hezhou, China; ^6^College of Life Science, Guangxi Normal University, Guilin, China

**Keywords:** wild and captive lizards, disease, diet, gut microbiota, *Shinisaurus crocodilurus*

## Abstract

The crocodile lizard is a critically endangered reptile, and serious diseases have been found in this species in recent years, especially in captive lizards. Whether these diseases are caused by changes in the gut microbiota and the effect of captivity on disease remains to be determined. Here, we examined the relationship between the gut microbiota and diet and disease by comparing the fecal microbiota of wild lizards with those of sick and healthy lizards in captivity. The gut microbiota in wild crocodile lizards was consistently dominated by Proteobacteria (∼56.4%) and Bacteroidetes (∼19.1%). However, the abundance of Firmicutes (∼2.6%) in the intestine of the wild crocodile lizards was distinctly lower than that in other vertebrates. In addition, the wild samples from Guangdong Luokeng *Shinisaurus crocodilurus* National Nature Reserve also had a high abundance of Deinococcus–Thermus while the wild samples from Guangxi Daguishan Crocodile Lizard National Nature Reserve had a high abundance of Tenericutes. The gut microbial community in loach-fed crocodile lizards was significantly different from the gut microbial community in the earthworm-fed and wild lizards. In addition, significant differences in specific bacteria were detected among groups. Notably, in the gut microbiota, the captive lizards fed earthworms resulted in enrichment of *Fusobacterium*, and the captive lizards fed loaches had higher abundances of *Elizabethkingia, Halomonas, Morganella*, and *Salmonella*, all of which are pathogens or opportunistic pathogens in human or other animals. However, there is no sufficient evidence that the gut microbiota contributes to either disease A or disease B. These results provide a reference for the conservation of endangered crocodile lizards and the first insight into the relationship between disease and the gut microbiota in lizards.

## Introduction

**FIGURE 1:**
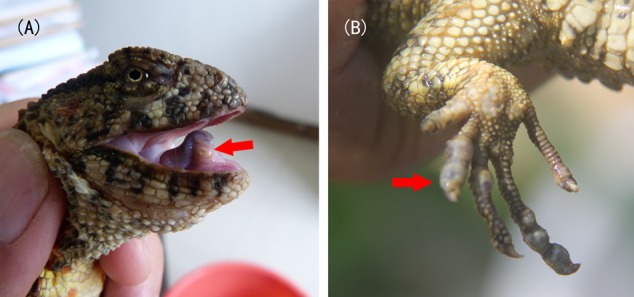
Symptoms of disease **(A)** and **(B)**. Arrows indicate the lesions.

The crocodile lizard (*Shinisaurus crocodilurus* Ahl, 1930) is the only species in the monotypic genus *Shinisaurus* and the monotypic family Shinisauridae. It is a relict reptile that now survives only in separated Pleistocene refugia. This species is distributed in southern China (Guangdong and Guangxi Provinces) and northern Vietnam (Qu
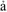
ng Ninh and Bac Giang Provinces) with severely fragmented populations (van Schingen et al., 2014, 2016). This species is essential not only for taxonomical systematics but also for understanding the origin, adaptation, and evolution of reptiles. However, it faces extinction due to the pressure of being hunted; environmental changes; and habitat destruction (Huang et al., 2008; Nguyen and Ziegler, 2015). It has been listed as an endangered species on the IUCN Red List of Threatened Species (Nguyen et al., 2014), a class I protected species in China, and an appendix I species by the Convention on International Trade in Endangered Species of Wild Fauna and Flora (CITES I). According to a recent survey, the total number of crocodile lizards in the wild has decreased from 6000 in 1978 to approximately 1200 in China (Huang et al., 2008; unpublished survey conducted by Wu et al., 2012). What’s worse, the population continues to decline sharply. Similarly, the wild population in Vietnam has decreased to fewer than 150 individuals in recent years (van Schingen et al., 2016). Some nature reserves, such as Guangdong Luokeng *S. crocodilurus* National Nature Reserve and Guangxi Daguishan Crocodile Lizard National Nature Reserve, are conducting captive breeding and release programs with the hope of restoring the wild populations. However, the captive individuals can become infected with serious diseases that cause many deaths each year (**Figure 1**). Crocodile lizards have two major types of diseases that can be distinguished by their symptoms. Disease A is characterized by one or more nodules in the underjaw or limbs covered by lesions (**Figure 1A**), while disease B is characterized by varying degrees of rot in the four limbs (**Figure 1B**). In the wild, individuals with disease B have been observed, while individuals with disease A have not been found.

Recent studies have revealed how variations and changes in the composition of gut microbial communities influence normal physiology and contribute to diseases (Clemente et al., 2012; Martin et al., 2014; Boursier et al., 2016). In addition, gut microbes affect host immunity, behavior, reproductive isolation, and metabolism (Cryan and Dinan, 2012; Brucker and Bordenstein, 2013; Ramakrishna, 2013; Thaiss et al., 2016; Sylvia et al., 2017). Conversely, many factors such as diet or host genetics can shape the microbial community (David et al., 2014; Goodrich et al., 2014).

Study on the gut microbiota has been conducted in a host of vertebrates, including mammals (Thaiss et al., 2016), birds (Hird et al., 2015; Waite and Taylor, 2015), fishes (Gajardo et al., 2016), amphibians (Bletz et al., 2016), and reptiles (Costello et al., 2010; Yuan et al., 2015; Kohl et al., 2016, 2017; Ren et al., 2016). However, the majority of these studies have been conducted in mammalian hosts. Surveys of the gut microbiota in reptiles, an ancient group with more than 10,000 extant species (Uetz et al., 2016), remain rare except in the case of some economically important species, such as snakes (Costello et al., 2010; Colston et al., 2015) and turtles (Huang and Zhang, 2013; Yuan et al., 2015). A few studies have been conducted on the gut microbiota of lizards, which represent about 60% of reptiles (Martin et al., 2010; Hong et al., 2011; Kohl et al., 2016, 2017; Ren et al., 2016). Nevertheless, the interaction between the gut microbiome and disease in reptiles remains unclear.

According to previous studies, the reptilian immune system differs from those of other vertebrates in several aspects (Zimmerman et al., 2010). Here, two questions are raised. Is the gut microbiota associated with disease susceptibility in crocodile lizards? How does cultivation shape the gut microbiome of crocodile lizards? To explore these questions and to facilitate the protection of this endangered species, we analyzed the amplicon-based microbiome of cloacal swab samples from crocodile lizards using 16S rRNA gene sequencing. We compared the gut microbiota of sick, healthy, captive, and wild lizards to identify the interactions among specific diseases, diets, and the gut microbiota in crocodile lizards.

## Materials and Methods

### Sample Collection

All samples were collected from Guangdong Luokeng *S. crocodilurus* National Nature Reserve (referred to as “Luokeng Nature Reserve” in the following sections) and Guangxi Daguishan Crocodile Lizard National Nature Reserve (referred to as “Daguishan Nature Reserve” in the following sections). Thirty crocodile lizards were separated into six groups, namely, the wild group from Luokeng Nature Reserve (WLK, *n* = 7), the healthy earthworm-fed group (NLK, *n* = 4), the sick earthworm-fed group with disease A (SLK, *n* = 5), the wild group from Daguishan Nature Reserve (WDG, *n* = 8), the healthy loach-fed group (NDG, *n* = 3), and the sick loach-fed group with disease B (SDG, *n* = 3). In addition, because of the highly similarity, the sick and healthy groups that fed the same diet were merged and recalculated. The sick and healthy individuals that fed earthworm were merged as earthworm-fed group (CLK), and the sick and healthy individuals that fed loach were merged as loach-fed group (CDG). Detailed sample information is shown in **Table 1**. Cloacal swabs were used for nondestructive sampling of the gut microbiota (Colston et al., 2015). The cloacal swabs were collected and stored in absolute ethyl alcohol or liquid nitrogen and then transported to the lab for DNA extraction within 24 h.

**Table 1:** Sample information used in this study.

Group	Sample	Health condition	Major diet	Location
WLK	WLK03	Healthy	Wild	Guangdong Luokeng *Shinisaurus crocodilurus* National Nature Reserve, Shaoguan City, Guangdong Province
	WLK06			
	WLK07			
	WLK08			
	WLK09			
	WLK10			
	WLK11			
WDG	WDG02	Healthy	Wild	Guangxi Daguishan Crocodile Lizard National Nature Reserve, Hezhou City, Guangxi province
	WDG03			
	WDG05			
	WDG06			
	WDG07			
	WDG08			
	WDG09			
	WDG10			
SLK (CLK)	SLK16	Infected with disease A (**Figure 1A**)	Captive, fed earthworms	Guangdong Luokeng *Shinisaurus crocodilurus* National Nature Reserve, Shaoguan City, Guangdong Province
	SLK17			
	SLK18			
	SLK19			
	SLK21			
NLK (CLK)	NLK22	Healthy	Captive, fed earthworms	Guangdong Luokeng *Shinisaurus crocodilurus* National Nature Reserve, Shaoguan City, Guangdong Province
	NLK23			
	NLK24			
	NLK25			
SDG (CDG)	SDG19	Infected with disease B (**Figure 1B**)	Captive, fed loaches	Guangxi Daguishan Crocodile Lizard National Nature Reserve, Hezhou City, Guangxi Province
	SDG31			
	SDG37			
NDG (CDG)	NDG28	Healthy	Captive, fed loaches	Guangxi Daguishan Crocodile Lizard National Nature Reserve, Hezhou City, Guangxi Province
	NDG35			
	NDG36			

All experimental animal procedures were approved by the Committee on the Ethics of Animal Experiments of the Guangdong Institute of Applied Biological Resources following basic principles.

### DNA Extraction and Sequencing

Total DNA was extracted from the cloacal swabs using a PowerFecal^®^ DNA Isolation Kit (MOBIO Laboratories, Inc., United States). The V4 hypervariable region of the 16S rRNA gene was amplified with the primers 515F (5′-GTGCCAGCMGCCGCGGTAA-3′) and 806R (5′-GGACTACHVGGGTWTCTAAT-3′), followed by library preparation using an NEB Next^®^ Ultra^TM^ DNA Library Prep Kit for Illumina (NEB, United States). Sequencing on an Illumina HiSeq platform (250 bp paired-end reads) was performed by Novogene Corporation (Beijing, China).

### Data Analysis

Raw tags were filtered using the QIIME V1.7.0 package (Caporaso et al., 2010) in order to remove the low-quality sequences and chimeras. Then, sequences with ≥97% similarity were assigned to the same operational taxonomic units (OTUs) using UCLUST in QIIME V1.7.0 package (Caporaso et al., 2010). A representative sequence for each OTU was annotated with threshold 0.8 using RDP Classifier 2.2 by searching the SILVA database (Wang et al., 2007; Quast et al., 2013).

For comparisons between samples, the OTU abundances were normalized by the number obtained from the sample with the lowest counts.

For each sample, alpha diversity was estimated by calculating the Shannon and abundance-based coverage estimator (ACE) indices. These indices were calculated by QIIME 1.7.0 (Caporaso et al., 2010) and displayed using R software. Alpha diversity indices were compared among samples using the Tukey method (*P* = 0.05) with R software.

Beta diversity was measured by principal coordinate analysis (PCoA) on unweighted and weighted UniFrac distances and were displayed using R software. The unweighted and weighted UniFrac distances were calculated by QIIME 1.7.0 (Caporaso et al., 2010). In addition, unweighted pair-group method with arithmetic means (UPGMA) clustering was also performed using QIIME 1.7.0 (Caporaso et al., 2010). The unweighted UniFrac distance accounts for membership in a community whereas the weighted UniFrac distance considers both membership and the relative abundance. Permutational multivariate analysis of variance (PERMANOVA) statistical analyses were conducted based on unweighted and weighted UniFrac distances with 999 permutations using function adonis in R’s vegan package.

To identify microbes accounting for the effects of disease and diet, the linear discriminatory analysis (LDA) effect size (LEfSe) method was used to compare the differential abundances of bacteria among groups at family and genus levels. LEfSe analysis emphasizes statistical significance, biological consistency, and effect relevance. It first robustly identifies taxa that are statistically different among groups. Then it investigates biological consistent using a set of pairwise tests among subgroups. At last, it uses LDA to estimate the effect size of each selected taxon. LEfSe analysis was performed using LEfSe software (Segata et al., 2011). The threshold of *P*-value in the Kruskal–Wallis test among groups was 0.05. Only those taxa with a log LDA score >4 (more than four orders of magnitude) were considered in this study.

All raw sequences obtained in this study have been deposited in the Sequence Read Archive (SRA) under accession number SRP107074.

## Results

We analyzed the bacterial composition of 30 crocodile lizard cloacal swab samples (**Table 1**). Each sample contained at least 30,000 effective sequences (Supplementary Figure 1). The rarefaction curves showed that these sequence depths were sufficient for capturing the major microbiota in each sample (Supplementary Figure 2). More than 99% of the OTUs could be well annotated at the family level in each sample (Supplementary Figure 3).

### General Pattern of the Gut Microbiota of the Wild Crocodile Lizard

**FIGURE 2:**
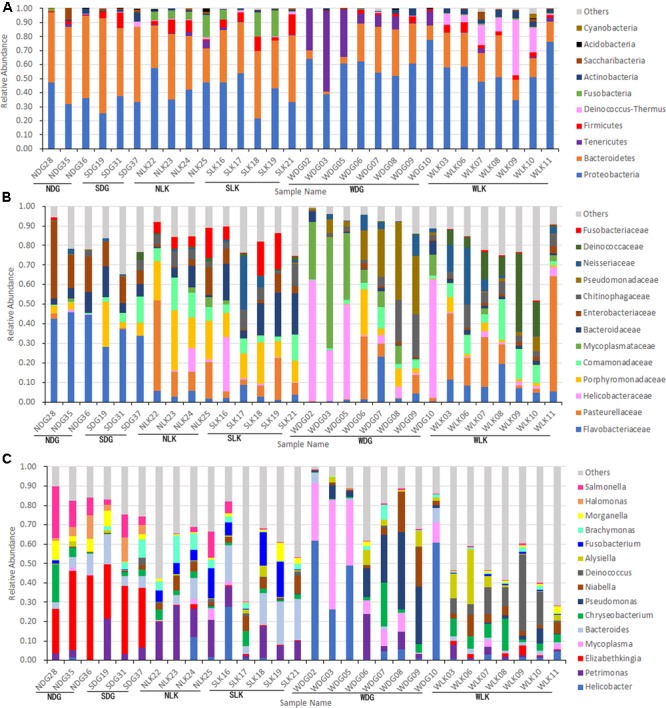
Composition of the gut microbiota of crocodile lizards at the phylum **(A)**, family **(B)**, and genus **(C)** levels.

In the total dataset, most of the bacteria were identified as Proteobacteria (47.9%) and Bacteroidetes (32.1%). At the phylum level, majority of species in the wild crocodile lizard gut microbiota were classified as Proteobacteria (56.4%), Bacteroidetes (19.1%), and Firmicutes (2.6%). In addition, the wild samples from Luokeng Nature Reserve also had a high abundance of Deinococcus–Thermus (13.6%) while the samples from Daguishan Nature Reserve had a high abundance of Tenericutes (5.9%). At the family level, the most abundant taxa in the wild crocodile lizard gut microbiota from Luokeng Nature Reserve were Pasteurellaceae, Deinococcaceae, Comamonadaceae, and Flavobacteriaceae. However, in Daguishan Nature Reserve, the wild crocodile lizard gut microbiota was dominated by Helicobacteraceae, Mycoplasmataceae, Pseudomonadaceae, and Chitinophagaceae. In Luokeng Nature Reserve, the most frequently occurring genera in the wild crocodile lizard intestine were *Niabella, Deinococcus, Alysiella*, and *Chryseobacterium.* In Daguishan Nature Reserve, the most frequently occurring genera in the wild crocodile lizard intestine were *Helicobacter, Mycoplasma, Pseudomonas*, and *Niabella*. However, the wild samples from Daguishan Nature Reserve were obviously separated into two patterns. Four samples had an extremely high abundance of *Mycoplasma* and *Helicobacter* (**Figure 2**).

**FIGURE 3:**
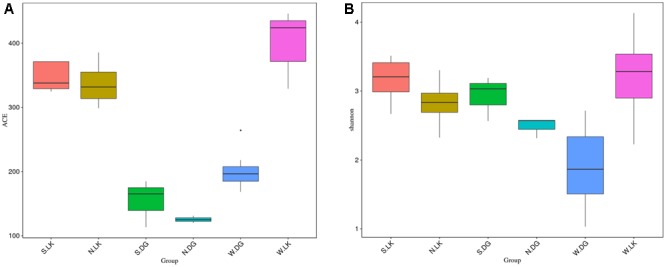
The alpha diversity of the gut microbial composition. **(A)** ACE index and **(B)** Shannon index. The bottom and top of the box are the first and third quartiles, the band inside the box is the median, and the ends of the whisker present the minimum and maximum.

The ACE and Shannon indices of the wild crocodile lizard gut microbiota from Luokeng Nature Reserve were significantly higher than that from Daguishan Nature Reserve (**Figure 3** and Supplementary Tables 1, 2). Therefore, both community richness and community diversity of the gut microbiota in wild crocodile lizards were different between distinct locations.

### Comparison of Gut Microbial Community Diversity between Groups

A comparison of alpha diversity indices between groups is presented in **Figure 3**. The significance between groups was detected by turkey method. For the community richness estimator (the ACE index), there was significant difference between earthworm-fed group and loach-fed group (NLK versus NDG). In addition, the loach-fed group was notably dissimilar with the wild group from the same place (NDG versus WDG), while the earthworm-fed group had no significant difference with the wild group from the same place (NLK versus WLK). However, there were no detectable significant differences between healthy and sick groups with the same diet. For the community diversity estimator (the Shannon index), no significant differences were found between these groups (**Figure 3** and Supplementary Tables 1, 2).

**FIGURE 4:**
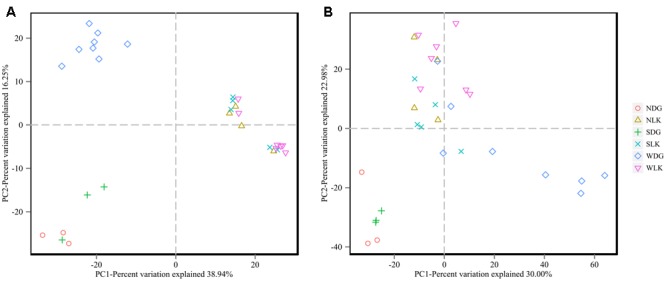
The beta diversity of the gut microbial composition. Principal coordinate analysis (PCoA) was conducted based on unweighted **(A)** and weighted **(B)** UniFrac distance matrices. The variation explained by the plotted principal coordinates is indicated in the axis labels.

**FIGURE 5:**
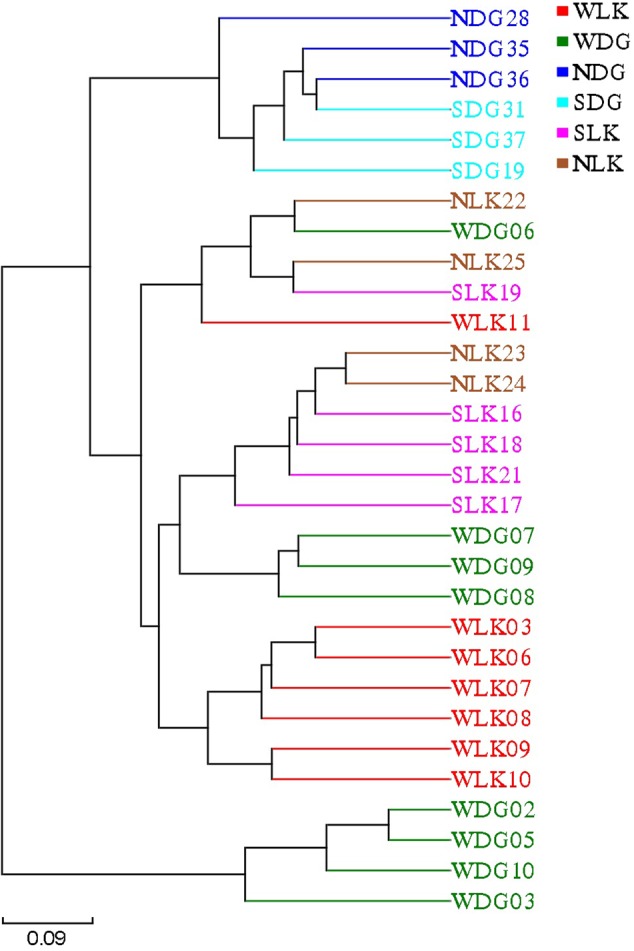
The beta diversity of the gut microbial composition. The UPGMA tree was generated based on weighted UniFrac distances.

The PCoA results also showed that samples from earthworm-fed group and loach-fed group were distantly separated for both community membership (**Figure 4A**) and community diversity (**Figure 4B**). In addition, the wild samples were separated from the loach-fed samples in Daguishan Nature Reserve. Nevertheless, for community diversity, four wild samples (WDG02, WDG03, WDG05, and WDG10) from Daguishan Nature Reserve were distantly separated from the other samples. These differences were also revealed by UPMA clustering (**Figure 5**). The separation of the four samples (WDG02, WDG03, WDG05, and WDG10) was resulted by the abnormally high abundances of *Mycoplasma* and *Helicobacter*, which decrease the community diversity. The earthworm-fed individuals were similar with the wild samples (**Figures 4, 5**). The sick and healthy groups overlapped for both community membership and community diversity (**Figures 4, 5**). In addition, the observed clusters were also supported by PERMANOVA analyses based on weighted and unweighted UniFrac metrics (*P* = 0.001, Supplementary Table 3).

Together with the results of alpha and beta diversity analyses, the gut microbiota in the sick and healthy groups was highly similar. Therefore, to well interpret the effect of diet, the sick and healthy samples that fed same diet were merged. The sick and healthy individuals that fed earthworm were merged as earthworm-fed group (CLK), and the sick and healthy individuals that fed loach were merged as loach-fed group (CDG). Then we recalculated the difference of alpha diversity indices between earthworm-fed group and loach-fed group. The results confirmed that the differences between groups of different diets were significant in terms of community richness (Supplementary Table 1).

In conclusion, there were significant differences in community richness and membership between groups of different diets. But there were no significant differences in the community diversity between any groups. However, no significant differences were found between healthy and sick groups in both community richness and community diversity.

### Differential Microbes among Groups

**FIGURE 6:**
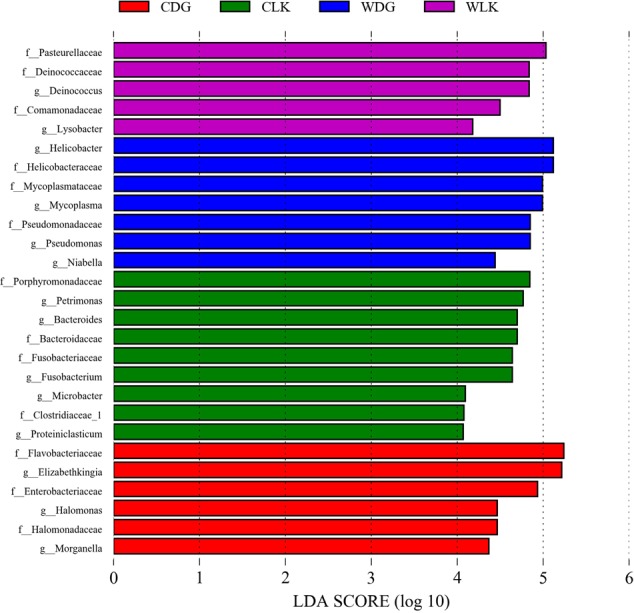
Differences in bacterial taxa among groups determined by linear discriminative analysis effect size (LEfSe). The highlighted taxa were significantly enriched in the group that corresponds to each color. LDA scores can be interpreted as the degree of difference in relative abundance. Abbreviation: g_, genus and f_, family.

**FIGURE 7:**
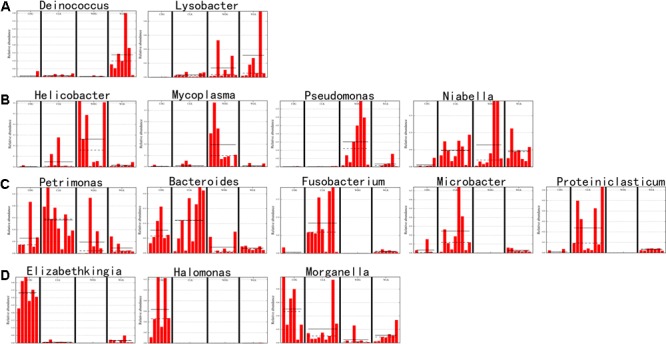
Relative abundances of differential microbes among groups. The taxa were selected by LEfSe analysis at the genus level. The straight line is the mean. The dot line is the median. **(A)** Dominant bacteria in the gut of wild crocodile lizards from Luokeng Nature Reserve. **(B)** Dominant bacteria in the gut of wild crocodile lizards from Daguishan Nature Reserve. **(C)** Dominant bacteria in the gut of captive crocodile lizards fed earthworms. **(D)** Dominant bacteria in the gut of captive crocodile lizards fed loaches.

The LEfSe analysis was used to screen the differential microbes among groups. Fourteen genera and 13 families were significantly enriched in distinct groups (**Figure 6**). The relative abundance of each selected genus is presented in **Figure 7**.

**FIGURE 8:**
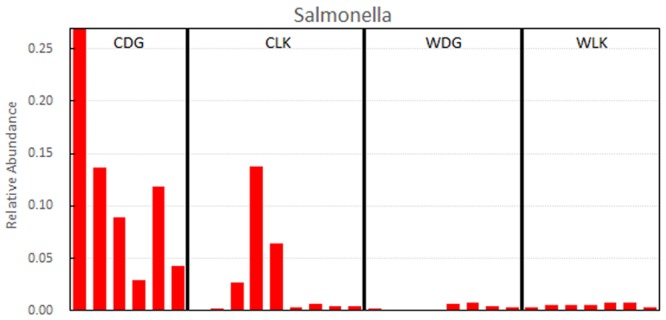
Relative abundances of *Salmonella*.

When compared the effects of diet on the crocodile lizard gut microbiota, the earthworm-fed group (CLK) showed increased abundances of genera *Petrimonas, Bacteroides, Fusobacterium, Microbacter*, and *Proteiniclasticum*, and families Porphyromonadaceae, Bacteriodaceae, Fusobacteriaceae, and Clostridiaceae. The loach-fed lizards (CDG) had a significant higher abundance of genera *Elizabethkingia, Halomonas*, and *Morganella*, and families Flavobacteriaceae, Enterobacteriaceae, and Halomonadaceae (**Figure 6**). When checking the relative abundance of each genus, *Salmonella* appeared in all samples, and significantly enriched in the gut of loach-fed lizards (**Figure 8**).

Compared with captive crocodile lizards, the gut microbiota of wild lizards from Luokeng Nature Reserve showed increased significantly in the abundances of genera *Deinococcus* and *Lysobacter*, and families Pasteurellaceae, Deinococcaceae, and Comamonadaceae. However, the wild lizards from Daguishan Nature Reserve had higher abundances of genera *Helicobacter, Mycoplasma, Pseudomonas*, and *Niabella*, and families Helicobacteraceae, Mycoplasmataceae, and Pseudomonadaceae (**Figure 6**).

## Discussion

Determining the role of the intestinal bacterial community in digestion and pathogenesis depends critically upon defining the “wild” and “normal” states. With high-throughput sequencing, it is now possible to comprehensively identify the bacteria in a given community, including fastidious and unculturable taxa. This study aimed to describe, for the first time, the wild state of the crocodile lizard gut microbiota as well as potential alterations in the composition of the gut microbiota of crocodile lizards with different diseases and diets compared with that in wild and healthy control subjects.

The composition of the gut microbiota in crocodile lizards is unique. Previous reports have indicated that the gut microbiota of lizards is dominated by the phyla Firmicutes (33.2–73%), Bacteroidetes (6.2–45.7%), and Proteobacteria (5.7–62.3%) (Martin et al., 2010; Nelson et al., 2010; Hong et al., 2011; Ren et al., 2016; Kohl et al., 2017). In other reptiles, the gut microbiota also appeared to be consistently dominated by Firmicutes, followed by Bacteroidetes, while Proteobacteria ranged from dominant to minor components (Costello et al., 2010; Colston et al., 2015; McLaughlin et al., 2015; Yuan et al., 2015). An exception is the alligator gut microbiota, which was dominated by Fusobacteria (Keenan et al., 2013). In other vertebrates, Firmicutes and Bacteroidetes also represent overwhelming majority of the gut microbiome (summarized by Keenan et al., 2013). Conversely, in the crocodile lizard gut microbiota, the proportion of Firmicutes was low, in all samples (0.13–14.56%), while Proteobacteria and Bacteroidetes were dominant (**Figure 2**). Moreover, in the wild samples from Luokeng Nature Reserve, the high prevalence of phylum Deinococcus–Thermus in the gut microbiome has not been found in other vertebrates. Deinococcus–Thermus spp. are usually found in extreme environments (Theodorakopoulos et al., 2013). They have also been detected in the feces of some animals with only a few clones (Lagier et al., 2012; McLaughlin et al., 2012). The role of Deinococcus–Thermus in the intestinal tract is not clear. However, Deinococcus–Thermus bacteria have been reported to show remarkable resistance to a range of stresses such as ionizing radiation, UV radiation, oxidizing agents, and desiccation (Theodorakopoulos et al., 2013). In addition, the relative abundance of Deinococcus–Thermus was significantly higher in the wild group than in the captive groups from the same place (**Figures 2, 7**). Therefore, the high proportion of Deinococcus–Thermus may help crocodile lizards adapt to the wild environment in Luokeng Nature Reserve. The wild samples from the Daguishan Nature Reserve had high abundances of *Mycoplasma* and *Helicobacter*. Particularly, the total abundances of *Mycoplasma* and *Helicobacter* reached up to 71.2–91.4% in samples WDG02, WDG03, WDG05, and WDG10. Because *Mycoplasma* and *Helicobacter* were detected in all samples, they are likely commensal inhabitants of crocodile lizards. *Mycoplasma* is a genus of bacteria that lack a cell wall and is primarily obligate commensals or parasites. Some species of *Mycoplasma* are significant pathogens of birds, mammals, fish, and reptiles, although many of them are harmless commensals to the hosts (Bano et al., 2007; Ossiboff et al., 2015). The overwhelming dominance of *Mycoplasma* have been reported in the fish gut or oyster stomach (Bano et al., 2007; King et al., 2012). However, the dominance of *Mycoplasma* in reptile guts was first reported. In the terms of *Helicobacter*, the most widely known member is *H. pylori*, which is strongly associated with peptic ulcers, chronic gastritis, duodenitis, and stomach cancer in humans (Khalifa et al., 2010). However, it is unclear whether the dominant *Mycoplasma* and *Helicobacter* would be harmful for the crocodile lizards.

Many studies showed that the gut microbiota had a relationship with a variety of diseases. In crocodile lizards, there was no significant correlation between the two diseases (A and B) and the gut microbiota. This may be because both disease A and B are located on the skin surface and do not have direct contact with the gut system.

Previous studies have shown that captivity can change the diversity of the gut microbiota (Nelson et al., 2013; Kohl and Dearing, 2014). In a study of *Anolis sagrei* insectivorous lizards also demonstrated that captivity led to a shift in microbial diversity (Ren et al., 2016). However, an investigation of omnivorous and herbivorous lizards showed that captivity had no significant effect on gut microbial diversity in terms of alpha diversity (Kohl et al., 2017). In this study, a comparison of alpha diversity indices, PCoA analysis, and UPGMA cluster suggested that the effect of captivity in shaping of gut microbiota was depend on diet. The diet of loach significantly changed the community richness of the gut microbiota of crocodile lizards but the diet of earthworm did not. However, captivity had no effect on the community diversity of gut microbiota of crocodile lizards according to the Shannon index.

The effect of captivity in shaping of gut microbiota was also reflected in the enrichment of specific microbes. The earthworm-fed group exhibited a gut microbiota enriched in *Petrimonas, Bacteroides, Fusobacterium, Microbacter*, and *Proteiniclasticum* compared with the wild and loach-fed groups (**Figures 6, 7**). Moreover, the captive lizards were consistently had a high abundance of *Bacteroides* compared with the wild samples (**Figure 7**). *Petrimonas* and *Microbacter* are fermenters isolated from environment (Grabowski et al., 2005; Sánchez-Andrea et al., 2014). The *Proteiniclasticum*, a genus of Clostridiaceae, is known as polysaccharide degrader in the gut (Zhang et al., 2010; Wust et al., 2011). A high relative abundance of Clostridiaceae was also observed in *Anolis* lizards (Hong et al., 2011). *Proteiniclasticum* may facilitate energy consumption in crocodile lizards. Notably, the diet of earthworm resulted in enrichment of *Fusobacterium*, which has been reported as human and animal pathogens (Signat et al., 2011). In addition, *Fusobacterium* has been commonly isolated from infected reptiles (Stewart, 1990). In the gut microbiota of other lizards, the abundance of *Fusobacterium*, if any, was very low, similar to that of the wild group of crocodile lizards (Martin et al., 2010; Hong et al., 2011; Kohl et al., 2016, 2017; Ren et al., 2016).

The loach-based diet resulted in the significant predominance of *Elizabethkingia, Halomonas, Morganella*, and *Salmonella* in the crocodile lizard gut microbiota. These genera are pathogens or opportunistic pathogens in animals and/or humans and have also been found in disease cases in reptiles (O’Hara et al., 2000; Bernardet et al., 2006; Stevens et al., 2009; Kim et al., 2013; Yeo et al., 2016). Particularly, attention should be paid to *Salmonella*, which causes enteritis and typhoid fever in mammalian and avian species. The *Salmonella* was detected in all samples, and its relative abundance in loach-fed crocodile lizards reached up to an average of 11.42% (**Figure 8**). Moreover, the zoonotic potential of *Salmonella* in reptiles has been widely documented (Murphy and Oshin, 2015). In addition, the *Salmonella* isolated from the focus of crocodile lizard with disease A caused the death of the Chinese skink in our lab (data have not been published). Moreover, *Elizabethkingia* was isolated from the focus of crocodile lizard with disease B (data have not been published). Therefore, hand washing should be recommended after contact with crocodile lizards, especially for those who contact these animals frequently. Although these genera have been detected as normal flora in many reptiles, there are many reports that associate these bacteria with reptile diseases such as bacterial pneumonia, osteomyelitis, septicemia, and hepatitis (Huchzermeyer, 1991; Ramsay et al., 2002; Grupka et al., 2006; Chinnadurai and Devoe, 2009). The high content of potential pathogenic bacteria in loach-fed group suggests that the diet of loach not only altered the structure of gut microbiota but also increased the risk of infection for crocodile lizards.

## Conclusion

The composition of the crocodile lizard gut microbiota is unique compared with other animals. Diets altered the bacterial community richness and the relative abundance of certain bacteria in the intestine. The gut microbiota of loach-fed crocodile lizard was significantly different from the gut microbiota of the wild and the earthworm-fed crocodile lizards. The earthworm-fed crocodile lizards had a higher abundance of *Fusobacterium* in the gut compared with the wild lizards. The intestine of loach-fed crocodile lizard was enriched in *Elizabethkingia, Halomonas, Morganella*, and *Salmonella.* These bacteria were reported to be pathogens or opportunistic pathogens in human or other animals. This may be a consequence of unbalanced nutrition, as the crocodile lizards in this study were routinely fed with only earthworms or loaches at the Nature Reserves. It seems that the diet of loach was not suitable for crocodile lizards. However, there is no sufficient evidence that the gut microbiota contributes to either disease A or disease B.

This study provides an overview of the gut microbiota of the crocodile lizard, an extremely endangered lizard, at different states as well as the first examination of the relationship between disease and the gut microbiota in lizards. These findings have numerous implications for the practice of crocodile lizard conservation, from the perspectives of both captivity and disease prevention. For instance, the results emphasize that a more diverse diet could improve the care of crocodile lizards in wildlife rescue centers and nature reserves. In addition, the diet of earthworm is better for crocodile lizard than the diet of loach.

## Ethics Statement

This study was carried out in accordance with the recommendations of Guidelines of Animal Experiments, the Committee on the Ethics of Animal Experiments of the Guangdong Institute of Applied Biological Resources. The protocol was approved by the Committee on the Ethics of Animal Experiments of the Guangdong Institute of Applied Biological Resources.

## Author Contributions

JPC and RCH designed the research. HYJ, NH, HYL, SYL, and ZJW collected the samples. HYJ, LML, and XJZ conducted the research. HYJ, JEM, and JL analyzed the data. HYJ and JPC wrote the manuscript. All authors approved the final version of the manuscript.

## Conflict of Interest Statement

The authors declare that the research was conducted in the absence of any commercial or financial relationships that could be construed as a potential conflict of interest. The reviewers AM and KK, and handling Editor declared their shared affiliation, and the handling Editor states that the process nevertheless met the standards of a fair and objective review.
