# Supplementary material for: Diets Alter the Gut Microbiome of Crocodile Lizards
Source: Front Microbiol. 2017 Oct 25;8:2073. doi: 10.3389/fmicb.2017.02073 (PMC5660983; doi:10.3389/fmicb.2017.02073)
Supplement: Supplementary file 1 [file Presentation_1.PDF]

Supplementary Material

Diets Shaping the Gut Microbiota of Crocodile Lizard

Hai-Ying Jiang, Jing-E Ma, Juan Li, Xiu-Juan Zhang, Lin-Miao Li, Nan He, Hai-Yang Liu, Shu-Yi Luo, Zheng-Jun Wu, Ri-Chou Han and Jin-Ping Chen*

*Correspondence: Jin-Ping Chen: chenjp@giabr.gd.cn

Supplementary Table 1. Comparison of the abundance-based coverage estimator (ACE) index between any two groups using Turkey's test.

Paired comparison	Mean difference	95% confidence interval		*P*	Significance
		Lower limit	Upper limit		
NDG-NLK	-211.539	-294.914	-128.163	0.000	*
NDG-SDG	-29.065	-118.197	60.068	0.911	
NDG-SLK	-221.485	-301.208	-141.763	0.000	*
NDG-WDG	-76.614	-150.518	-2.709	0.039	*
NDG-WLK	-276.336	-351.667	-201.006	0.000	*
NLK-SDG	182.474	99.099	265.850	0.000	*
NLK-SLK	-9.947	-83.176	63.283	0.998	
NLK-WDG	134.925	68.076	201.774	0.000	*
NLK-WLK	-64.798	-133.220	3.625	0.071	
SDG-SLK	-192.421	-272.143	-112.698	0.000	*
SDG-WDG	47.549	-26.355	121.454	0.377	
SDG-WLK	-247.272	-322.602	-171.941	0.000	*
SLK-WDG	144.871	82.638	207.105	0.000	*
SLK-WLK	-54.851	-118.771	9.069	0.123	
WLK-WDG	199.722	143.225	256.220	0.000	*
After NLK and SLK were merged into CLK, NDG and SDG were merged into CDG					
CDG-WDG	-62.082	-113.572	-10.592	0.014	*
CDG-WLK	-261.804	-314.847	-208.761	0.000	*
CDG-CLK	-202.532	-252.781	-152.283	0.000	*
CLK-WDG	140.451	94.123	186.778	0.000	*
CLK-WLK	-59.272	-107.319	-11.224	0.011	*
WLK-WDG	199.722	150.379	249.066	0.011	*

Supplementary Table 2. Comparison of the Shannon index between any two groups using Turkey's test.

Paired comparison	Mean difference	95% confidence interval		*P*	Significance
		Lower limit	Upper limit		
NDG-NLK	-0.337	-1.521	0.848	0.948	
NDG-SDG	-0.441	-1.708	0.825	0.886	
NDG-SLK	-0.670	-1.802	0.463	0.468	
NDG-WDG	0.579	-0.471	1.629	0.542	
NDG-WLK	-0.728	-1.798	0.342	0.319	
NLK-SDG	-0.105	-1.289	1.080	1.000	
NLK-SLK	-0.333	-1.373	0.707	0.917	
NLK-WDG	0.915	-0.034	1.865	0.063	
NLK-WLK	-0.391	-1.363	0.581	0.811	
SDG-SLK	-0.228	-1.361	0.904	0.988	
SDG-WDG	1.020	-0.030	2.070	0.060	
SDG-WLK	-0.287	-1.357	0.784	0.959	
SLK-WDG	1.248	0.364	2.133	0.003	*
SLK-WLK	-0.058	-0.967	0.850	1.000	
WLK-WDG	1.307	0.504	2.109	0.000	*
After NLK and SLK were merged into CLK, NDG and SDG were merged into CDG					
CDG-WDG	0.7994792	0.054329	1.544629	0.032	*
CDG-WLK	-0.5072762	-1.274898	0.260346	0.29	
CDG-CLK	-0.3009333	-1.028125	0.426259	0.672	
CLK-WDG	1.1004125	0.429975	1.77085	0.001	*
CLK-WLK	-0.2063429	-0.901672	0.488986	0.847	
WLK-WDG	1.3067554	0.592667	2.020844	0	*

Supplementary Table 3. PERMANOVA results based on weighted and unweighted UniFrac distance matrices

Distance matrices	Source of variation /Pairwise comparison	d.f.	SS	*F*	R^2	*P*	Significance
Weighted UniFrac distance matrices	Group	5	3.174	6.051	0.558	0.001	*
	Residuals	24	2.518		0.442		
	Total	29	5.692		1.000		
	NDG-NLK			25.017	0.833	0.027	*
	NDG-SDG			0.893	0.183	0.500	
	NDG-SLK			16.232	0.730	0.023	*
	NDG-WDG			5.173	0.365	0.015	*
	NDG-WLK			15.909	0.665	0.010	*
	NLK-SDG			21.805	0.813	0.036	*
	NLK-SLK			0.917	0.116	0.489	
	NLK-WDG			5.164	0.360	0.012	*
	NLK-WLK			6.606	0.423	0.004	*
	SDG-SLK			13.643	0.695	0.019	*
	SDG-WDG			4.730	0.345	0.022	*
	SDG-WLK			14.076	0.638	0.012	*
	SLK-WDG			4.369	0.284	0.012	*
	SLK-WLK			6.226	0.384	0.003	*
	WDG-WLK			4.501	0.257	0.036	*
Unweighted UniFrac distance matrices	Group	5	2.421	6.137	0.561	0.001	*
	Residuals	24	1.893		0.439		
	Total	29	4.314		1.000		
	NDG-NLK			11.951	0.705	0.027	*
	NDG-SDG			1.210	0.232	0.300	
	NDG-SLK			10.375	0.634	0.013	*
	NDG-WDG			10.395	0.536	0.007	*
	NDG-WLK			16.248	0.670	0.012	*
	NLK-SDG			7.846	0.611	0.023	*
	NLK-SLK			0.125	0.018	0.956	
	NLK-WDG			22.893	0.696	0.001	*
	NLK-WLK			2.129	0.191	0.156	
	SDG-SLK			7.029	0.540	0.027	*
	SDG-WDG			6.610	0.423	0.008	*
	SDG-WLK			11.663	0.593	0.010	*
	SLK-WDG			21.877	0.665	0.003	*
	SLK-WLK			1.532	0.133	0.191	
	WDG-WLK			34.968	0.729	0.001	*

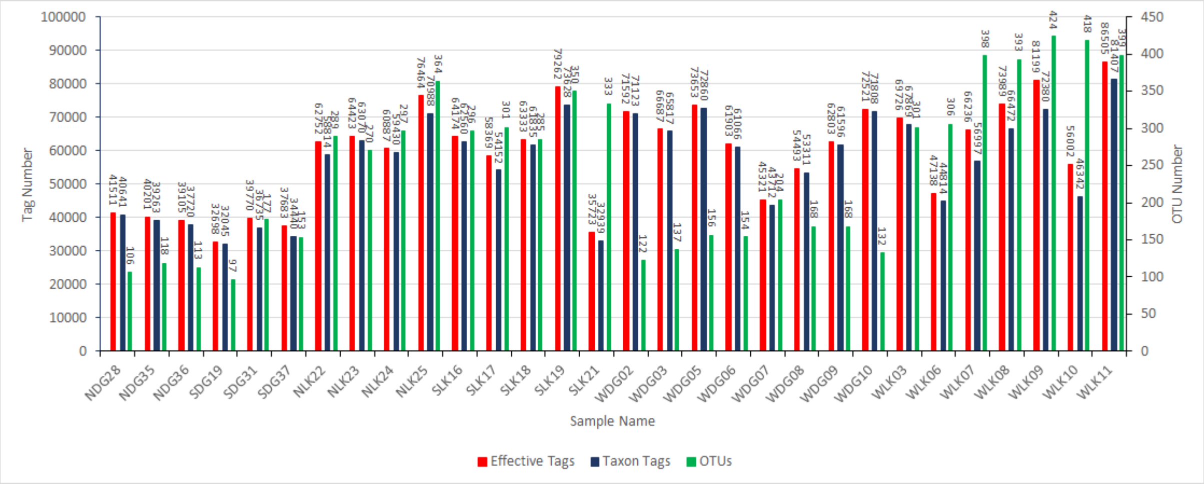

Supplementary Figure 1. Numbers of effective sequences and OTUs in each sample. Effective tags (red) were sequences without low-quality sequences and chimeras, and were used for annotation and other analyses. Taxon tags (blue) represent the sequences that could be clustered into OTUs and annotated.

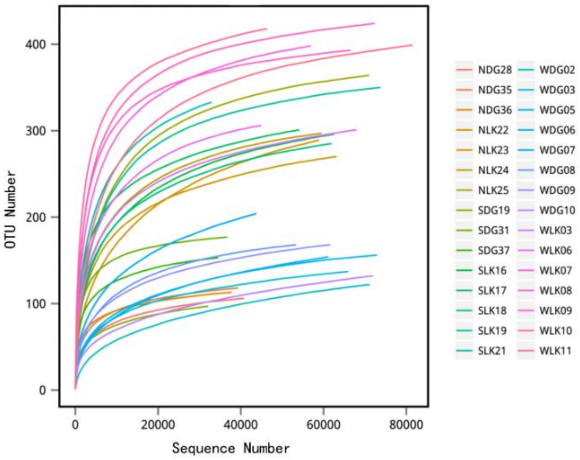

Supplementary Figure 2. Rarefaction curves of each sample.

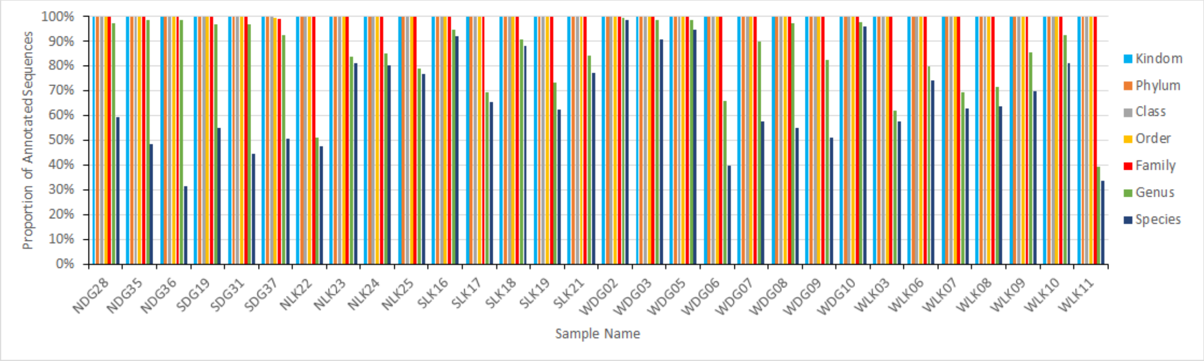

Supplementary Figure 3. Percentage of the annotated bacteria at different taxonomical levels.
